# The story of the Malagasy devils (Orthoptera, Tetrigidae): *Holocerus lucifer* in the north and *H. devriesei* sp. nov. in the south?

**DOI:** 10.3897/zookeys.957.52565

**Published:** 2020-08-10

**Authors:** Josip Skejo, Kristian Medak, Marko Pavlović, †Davorka Kitonić, Rafanomezanjanahary Jean Christian Miko, Damjan Franjević

**Affiliations:** 1 University of Zagreb, Faculty of Science, Department of Biology, Division of Zoology, Evolution Lab, Rooseveltov trg 6, HR-10000, Zagreb, Croatia University of Zagreb Zagreb Croatia; 2 SIGTET - Special Interest Group Tetrigidae, Croatia Special Interest Group Tetrigidae Zagreb Croatia; 3 Institute of Molecular Evolution, Heinrich - Heine University Düsseldorf, Germany Heinrich - Heine University Düsseldorf Düsseldorf Germany; 4 Crepina 44, HR-20355 Opuzen, Croatia (passed away on April 2nd 2020) Unaffiliated Opuzen Croatia; 5 Association Mitsinjo, Andasibe-Mantadia NP, Madagascar National Parks, Andasibe, Madagascar Madagascar National Parks Andasibe Madagascar

**Keywords:** pygmy grasshoppers, Tetrigoidea, Metrodorinae, *
Holocerus
*, Hendrik Devriese, Madagascar, new species, threatened species

## Abstract

Madagascar is home to some of the largest and most colorful pygmy grasshoppers (Tetrigidae) in the world, known as ‘Malagasy Metrodorinae’. Among them, Devil’s pygmy grasshoppers (genus *Holocerus* Bolívar, 1887) are unique in having two long spines on the back, which are modified internal lateral pronotal carinae. The genus *Holocerus* was composed of two species – *H. lucifer* (Serville, 1838) and *H. taurus* Rehn, 1929 **syn. nov.**, but here it is evidenced that the latter represents a junior synonym of the former. Simultaneously, *H. devriesei***sp. nov.** is described as a species new to science. *Holocerus lucifer* is a northern species of paler coloration and longer spines (distributed from Marojejy and Maroantsetra in the north to Zahamena in the south), whereas *H. devriesei***sp. nov**. represents the southern and darker species (distributed from Vohimana and Andasibe-Mantadia in the south to the Antongil Bay in the north). There are potential overlaps in the distribution of the two species, but without more georeferenced localities, it is impossible to discriminate whether they occur only sympatrically or also syntopically.

## Introduction

Research on Malagasy Tetrigoidea has, to date, been carried out for 180 years. Pioneer researchers, such as Serville (1838), Bolívar (1887) and Brancsik (1892), examined the material collected by researchers who took part in expeditions. A lot of research has been added to the knowledge of the pygmy grasshoppers of Madagascar since the time of the pioneers, and today we are aware of the existence of 75 species, most of which are endemic to the island (e.g., Bruner 1910; Devriese 1991; Günther, 1959; 1970; Hancock 1900, 1907).

Serville (1838) described three species of peculiar pronotal morphology and named them after Biblical demons – (i) *Tetrix asmodaeus* Serville, 1838 (now in *Pterotettix*), (ii) *T. belphegor* Serville, 1838 (now in *Pterotettix*), and (iii) *T. lucifer* Serville, 1838 (now in *Holocerus*). *Tetrix lucifer* has lateral pronotal carinae projected in two long spines, one on each side of the pronotum. Bolívar (1887) placed *T. lucifer* in the genus *Holocerus* Bolívar, 1887, which belongs to the likely monophyletic group of ‘Malagasy Metrodorinae’, also including *Andriana* Rehn, 1929, *Bara* Rehn, 1929, *Eurybiades* Rehn, 1929, *Hovacris* Rehn, 1929, *Hybotettix* Hancock, 1900, *Notocerus* Hancock, 1900, *Rehnitettix* Günther, 1939, and *Silanotettix* Günther, 1959 (Günther 1959, 1974). The group is characterized by (i) antennae with modified segments, (ii) projected or undulated median and internal lateral carinae of the pronotum, (iii) elevated promedial projection (a spine on the median carina of the pronotum in the prozona), (iv) lack of tegminal sinus, (v) rounded pulvilli of the hind tarsi, (vi) lack of humeral carinae, and (vii) rich coloration (Günther 1974; Devriese 1991). The longest spines are exhibited by members of the genera *Eurybiades*, *Holocerus*, and *Notocerus*, known as the Devil’s pygmy grasshoppers.

We know about the existence of two *Holocerus* species, as defined by Rehn (1929). Those are Serville’s *H. lucifer*, which is, according to Rehn, a darker species with shorter dorsal spines, and Rehn’s *H. taurus*, a yellowish-green species with long spines. After the insight into the entomological collections of the MNHN in Paris and the ANSP in Philadelphia, it became evident that both Serville and Rehn described the very same species. In this study, we provide photographs of *Tetrix lucifer* and *Holocerus taurus* type specimens, as well as living specimens, and we aim to clarify once and for all what the epithet ‘*lucifer*’ refers to. We present an annotated distribution of *H. lucifer*, a pale colored species with long spines; synonymization of *H. taurus* Rehn, 1929 syn. nov. with *H. lucifer*; and an annotated distribution of a newly described darker species with shorter spines, *H. devriesei* sp. nov.

## Materials and methods

### Acronyms of museum collections

**ANSP** – The Academy of Natural Sciences of Drexel University, Philadelphia, USA; **JSTC** – Josip Skejo Tetrigidae Collection, Zagreb, Croatia; **MNCN** – Museo Nacional Ciencias Naturales, Madrid, Spain; **MNHN** – Muséum national d’Histoire naturelle, Paris, France.

### Abbreviations

**HT** – holotype; **OSF** – Orthoptera Species File (Online Database of Orthoptera – Cigliano et al. 2020); **PT** – paratype; **PTs** – paratypes.

### Identification, nomenclature and taxonomy

The two taxa within the genus *Holocerus* can be easily distinguished based on the characteristics presented by Rehn (1929). However, Rehn’s nomenclature is incorrect. The two could be treated as species or subspecies, but based on the overlaps in their distributions and no evidence of intermediate forms, we treated them as separate species. Nomenclature follows the International Code of Zoological Nomenclature (ICZN 1999), whereas Tetrigidae taxonomy follows the Orthoptera Species File (Cigliano et al. 2020).

### Morphological terminology and measurements

We followed Tumbrinck (2014) for the description of general morphology; Devriese (1991, 1995, 1999) for the nomenclature of pronotal carinae; and Pushkar for terminology of pronotal projections (Storozhenko and Pushkar 2017). Measurements were taken on museum specimens in ImageJ2 (Rueden et al. 2017) on the traits previously measured in *Holocerus* taxonomy (Rehn 1929). The accuracy of the measurements was 0.1 mm.

### Photography

Several photos of living individuals were obtained online. These are included in the study with the permission of the photographers (Rowe Becky, Paul Bertner, Marc Hoffmann, and Frank Vassen).

## Results

### Taxonomic treatment

#### Family Tetrigidae Rambur, 1838

**Subfamily Metrodorinae Bolívar, 1887**

**Informal group, ‘Metrodorinae of Madagascar**’

**Composition and tentative diagnosis.** The group is composed of the genera *Andriana* (4 sp.), *Bara* (1 sp.), *Eurybiades* (1 sp.), *Holocerus* (2 spp.), *Hovacris* (1 sp.), *Hybotettix* (2 spp.), *Notocerus* (2 spp.), *Rehnitettix* (1 sp.), and *Silanotettix* (3 spp.), which share the lack of a tegminal sinus; the lack of humeral carinae; rounded tarsal pulvilli; modified antennal segments; and pronotum with projected and undulated parts (Devriese 1991).

##### Genus Holocerus

Taxon classificationAnimaliaOrthopteraTetrigidae

Bolívar, 1887

25F7A707-A3A4-5C96-813E-32AD15BDD369

Tetrix Latreille, 1802 (partim): Serville (1838: 758); Augé (1898: 296; first depiction of Holocerus lucifer after the holotype);
Holocerus Bolívar, 1887: Bolívar (1887: 186, 231–232; tentative description, assignment to Metrodorinae); Kirby (1910: 28; listed in catalogue); Rehn (1929: 492–493; redescription); Rehn (1937: 320; new records); Günther (1939: 91; listed in catalogue, taxonomic position discussed); Günther (1959: 11; included in key, discussed), Günther (1970: 79–92; discussed); Devriese (1995: 123–124; mentioned and depicted); Yin et al. (1996: 876; listed in catalogue); Otte (1997: 45; listed in catalogue); Skejo and Caballero (2016: figs 2a, b; mentioned and depicted); Skejo (2017: 14, 19, 68; listed in catalogue); Cigliano et al. (2020; OSF catalogue).

###### Type species.

*Tetrix lucifer* Serville, 1838 (*Holocerus lucifer*), by monotypy (Bolívar).

###### Composition and distribution.

The genus is composed of two species, *Holocerus lucifer* and *H. devriesei* sp. nov. Both species inhabit rainforests of East Madagascar, from Ranomafana in the south to Marojejy in the north.

###### Ecology and habitat.

Records of adults and nymphs in different parts of the year indicate that the species may be active throughout the year. Devil’s pygmy grasshoppers are rainforest dwellers and they inhabit primary and secondary rainforests of Madagascar, where they can be found standing on both wet and dry tree bark of species such as the traveler’s palm (*Ravenala madagascariensis*; order *Zingiberales*: family *Strelitziaceae*) (Figure 4). *Holocerus lucifer* and *H. devriesei* sp. nov. are good fliers and can glide for long distances (> 10 m) between rainforest trees and bushes.

###### Generic diagnosis and affinity to other genera.

The genus *Holocerus* is similar to the genera *Notocerus* (2 spp.) and *Eurybiades* (1 sp.), which are both endemic to Madagascar. Unlike the clearly separated dorsal spines in *Holocerus*, members of the genus *Notocerus* (*N. cornutus* Hancock, 1900 and *N. formidabilis* Günther, 1974) have a highly elevated dorsum between the spines (making the spines connected). With the morphology of its spines, *Notocerus cornutus* is more similar to the members of the genus *Holocerus* than to its own congener, *N. formidabilis* (which has a high and warty hump). *Holocerus* can also easily be distinguished from *Notocerus* by the more prominent eyes. The only species from the genus *Eurybiades*, *E. cerastes* Rehn, 1929, is much smaller than the members of the genus *Holocerus*, and is easily distinguished from both *Holocerus* and *Notocerus* members by the long promedial projection, which is spiky and directed forwards. *Holocerus* and *Notocerus* have a short and tooth-like promedial projection of the prozona.

##### Holocerus lucifer

Taxon classificationAnimaliaOrthopteraTetrigidae

(Serville, 1838)

33B198D6-079E-5E64-82E6-464B852D4AD2

**Figure 1.**
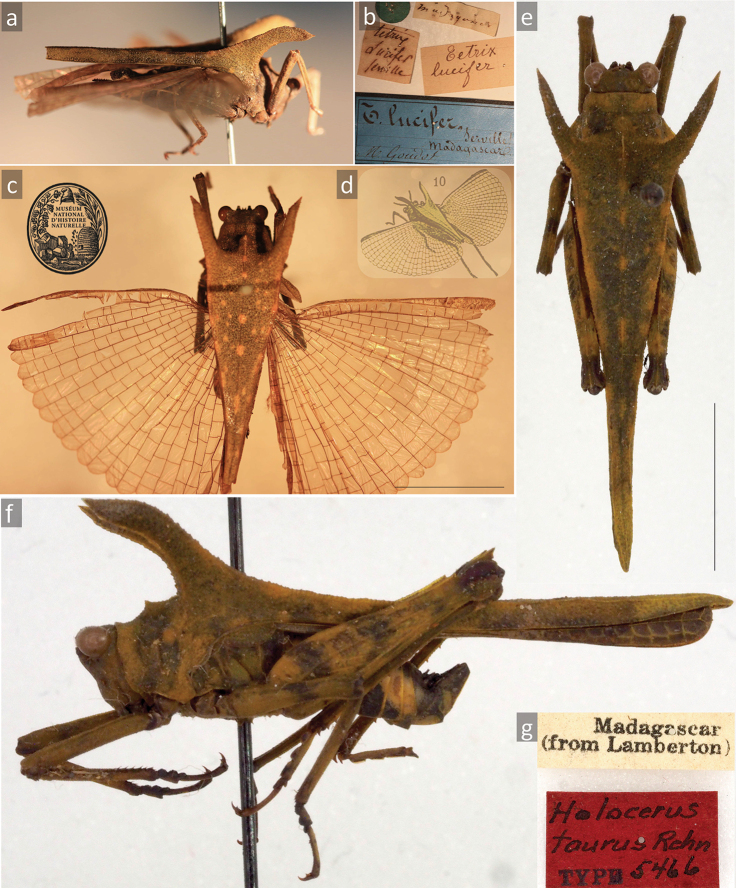
**A–F***Holocerus lucifer*. Female holotype of *Holocerus lucifer* (original combination *Tetrix lucifer*) from MNHN Paris (**A–D**) and male holotype of *Holocerus taurus* syn. nov. from ANSP Philadelphia (**E–G**) **A** habitus in lateral view **B** labels **C** habitus in dorsal view, (photos **A–D** J. Skejo & MNHN Paris) **D** the first depiction of *Holocerus lucifer* (Augé 1898) **E–G** holotype of *Holocerus taurus* (from here on syn. nov. of *H. lucifer*) **E** habitus in dorsal view **F** habitus in lateral view **G** labels (photos **E–G** Jason D. Weintraub / ANSP Entomology). Scale bar: 1 cm.

**Figure 2.**
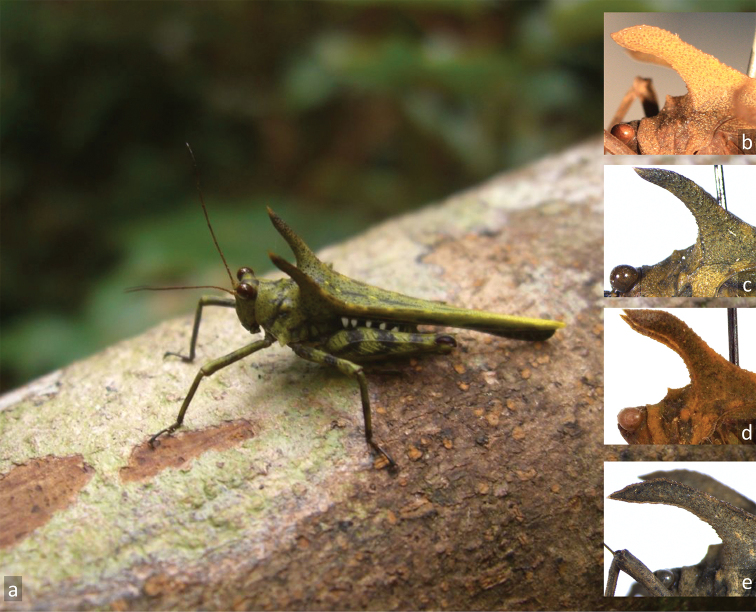
Variability of *Holocerus lucifer*. **A** living specimen in Marojejy NP (photo R. Becky) **B–E** variability of pronotal projection morphology (**B** holotype of *Holocerus lucifer***C** Maroantsentra, Antongil Bay **D** holotype of *H. taurus***E** Tamatave).

Figs 1 , 2 Common name: Northern Devil’s pygmy grasshopper

Tetrix Lucifer Serville, 1838: Serville (1838: 758; description of the species based on a single ♀ holotype (MNHN) from Paris, see Fig. 1A–D); Augé (1898: 296; drawing of the HT from MNHN Paris = first depiction of this genus member, Fig. 1D);
Holocerus Lucifer : Bolívar (1887: 186, 231, 232; mentioned, tentatively assigned to the genus Holocerus); Kirby (1910: 28; listed in catalogue);
Holocerus taurus Rehn, 1929 syn. nov.: Rehn (1929: 494–497; detailed description of the species based on seven specimens, ♂ HT, 1 ♀ PT and 3 ♂♂ PTs from unknown locality, and 2 ♀♀ PTs from Maroantsetra, depicted) (Fig. 1E–G); Günther (1939: 91; mentioned, new records); Günther (1959: 11; included in key, depicted); Günther (1970: 79–92; discussed); Günther (1974: 966, 967; new records, depiction); Yin et al. (1996: 876; listed in catalogue); Otte (1997: 45; listed in catalogue); Cigliano et al. (2020; listed in catalogue).

###### Type material.

(1 HT of *T. lucifer* (Figure 1A–D), 1 HT (Figure 1E–G) + 6 PTs of *H. taurus*). ♀ HT of *Tetrix lucifer*, Madagascar (MNHN); ♂ HT of *Holocerus taurus*, Madagascar, 1♀ PT from Madagascar, 3♂♂ PTs from Madagascar, 2♀♀ PTs from Maroantsetra II.1919 (ANSP).

###### Additional examined material.

There are a few records of the species since its description – by Rehn (1929) under the name *H. taurus*; a photographic record from Flickr; a photographic record from Twitter; and several specimens from a museum collection in MNCN, Madrid. For detailed data on all records, see Table 1.

**Table 1.** List of all known records of *Holocerus lucifer* with approximate coordinates and a reference or link for each record. (*in the narrow sense, Tamatave or Toamasina region probably refers to rainforests close to the city of Toamasina, whereas in the wide sense, this region covers most of the rainforests from the city of Toamasina to the Bay of Antongil).

	Locality	Coordinates	Date	References or link
**1**	no specified locality	N/A	N/A	1♀ HT of *Tetrix Lucifer*: Serville 1838: 758; Bolívar 1887: 186, 231, 232, Augé 1898: 296; Kirby 1914
**2**	no specified locality	N/A	N/A	1♂ HT and 4 PTs (1♀, 3♂♂) of *Holocerus taurus*: Rehn 1929: 497
**3**	Antongil Bay: Maroantsetra	15.34S, 49.86E	II.1919.	2♀♀ PTs of *Holocerus taurus*: Rehn 1929: 497
**4**	Antongil Bay	15.34S, 49.86E	N/A	1♂, 1♀ (deposited in Vienna) (Günther 1939: 91)
**5**	Antongil Bay: Maroantsetra	15.34S, 49.86E	N/A	3♂♂ 12.1897. (deposited in MNCN Madrid, MNCN_ Ent 268523, MNCN_Ent 268525 and MNCN_Ent 268525)
**6**	Marojejy: Ambonanitelo	15.374S, 49.523E	XII.1958.	2♀♀, 2♂♂, 1 nymph leg. E. Raharizonina (Günther 1974: 967)
**7**	Marojejy: Ambatosoratra	14.43S, 49.73E	VII.[19]60.	1 nymph leg. P. Soga (Günther 1974: 967)
**8**	Maroantsetra: Ambodivoangy	14.401S, 49.941E	III.[19]49.	1♀ leg. Michel (Günther 1974: 967)
**9**	Marojejy NP	14.437S, 49.742E	01.I.2006.	Living specimen, ID H. Devriese, photo Rowe_Becky, https://www.flickr.com/photos/rowe_becky/497874026
**10**	Zahamena NP	17.612S, 48.779E	X.2000.	1♀ (uploaded by Chris Grinter to Twitter)
**11**	Tamatave (= Toamasina) *	17.92S, 48.96E	N/A	1♂ 1888. Leg. Perrot (deposited in MNCN Madrid, MNCN_Ent 268526)

###### Annotated specific diagnosis.

*Holocerus lucifer* is similar to *H. devriesei* sp. nov., which is the only other species of the genus *Holocerus*. *Holocerus lucifer* is easily distinguished from *H. devriesei* sp. nov. with the following set of characteristics: (i) *Holocerus lucifer* has slenderer femora of fore and mid legs than that of *H. devriesei* sp. nov.; (ii) dorsal spines are slenderer, more elongate and decurved in *H. lucifer* than those in *H. devriesei* sp. nov., and in profile they are, as described by Rehn, ‘distinctly falcate, scimitar like’; (iii) middle prozonal spine (promedial projection) is blunter and lower in *H. lucifer* than that in *H. devriesei* sp. nov., and (iv) *H. lucifer* generally has more pale colored parts than *H. devriesei* sp. nov.

###### Measurements.

Body length, pronotum length, pronotum width, and hind femora length are shown (Table 2).

**Table 2.** *Holocerus lucifer* measurements (‘*taurus*’ is synonymous with ‘*lucifer*’, and these are the measurements of its type specimens). Note that the locality is known only for one individual (♀ from Maroantsetra).

	Body length	Pronotum length	Pronotum width (humeral spines)	Hind femora length
*Lucifer* ♀HT (Madagascar)	18.8 mm	>19.8 mm (tip broken)	8.4 mm	N/A (missing)
*taurus* ♂HT (Madagascar)	14.2 mm	22.3 mm	8.2 mm	10.0 mm
*taurus* ♂PT (Madagascar)	13.9 mm	20.0 mm	8.0 mm	9.0 mm
*taurus* ♀PT (Madagascar)	17.6 mm	22. 6 mm	N/A (broken)	10.6 mm
*taurus* ♀PT (Maroantsetra)	20.8 mm	23.2 mm	8.5 mm	11.2 mm

###### Variability.

(Figure 2). Variability is evident in (1) coloration, as there are darker and paler specimens, (2) size, as there are larger and smaller specimens, and (3) the shape of the dorsal spines (elevated internal lateral carinae of the pronotum), as there are specimens in which the spines are more decurved and specimens with almost straight projections.

###### Distribution and habitat.

The species is known from the rainforests of Madagascar, from Marojejy and Maroantsetra in the north to the rainforests of Zahamena in the south. A specimen with the label ‘Tamatave’ could have been collected in the rainforests in the vicinity of the city of Toamasina, but also anywhere in the wide Toamasina region. The species inhabits primary and secondary rainforests and is probably a good flier, taking into account the observations of its sibling species’ ecology. As only one photo of a living individual of this species has been taken to date, hardly anything can be concluded about the species’ natural history. Despite being described for already 180 years, this species is less known and understood than *H. devriesei* sp. nov.

##### Holocerus devriesei sp. nov.

Taxon classificationAnimaliaOrthopteraTetrigidae

34E1888B-A77F-5470-80DE-9A5930D84A9B

http://zoobank.org/4A0CCADC-D104-489D-A350-F4B595AC559B

**Figure 3.**
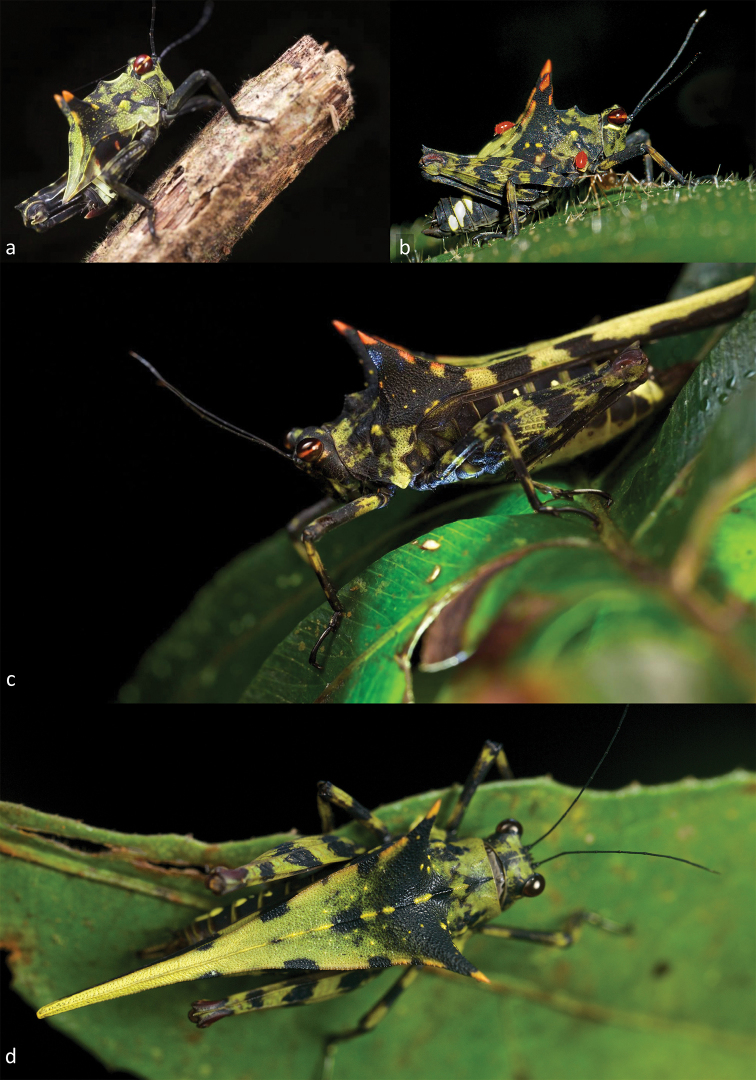
*Holocerus devriesei* sp. nov. in natural habitat. **A** Nymph from Andasibe (photo P. Bertner) **B** nymph from Vohimana (photo F. Vassen) **C** adult ♀ from Andasibe in c in dorsal view and **D** in dorsal view (photo P. Bertner).

**Figure 4.**
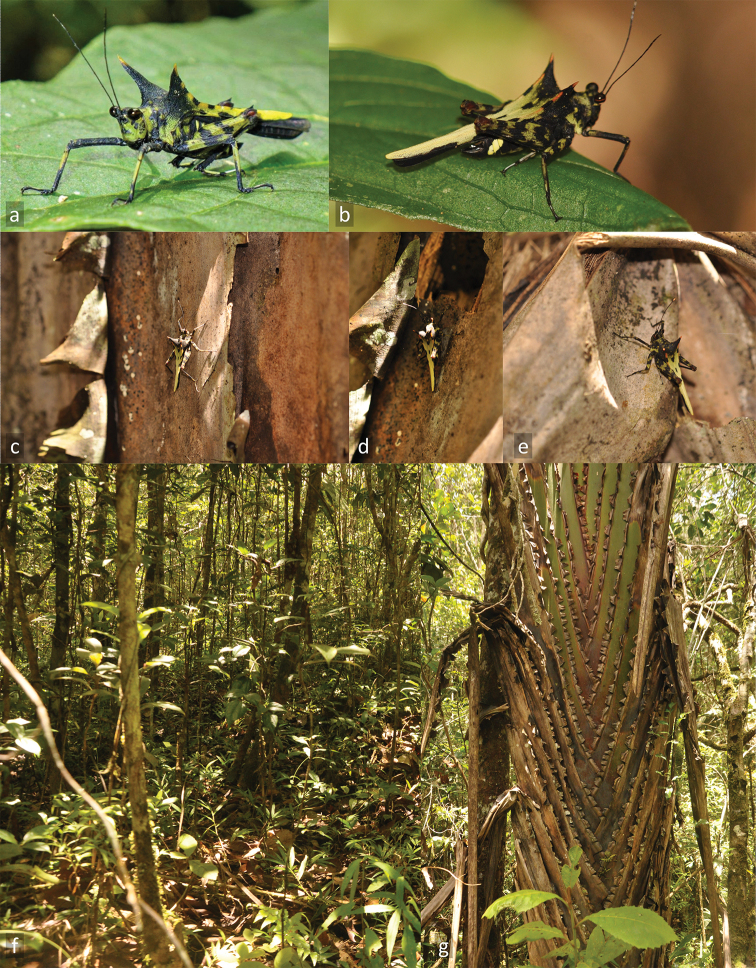
*Holocerus devriesei* sp. nov. and its habitat. **A** ♂ from Ranomafana in natural habitat (photo M. Hoffmann) **B–E** adult ♂ from Analamazaotra **F–G** natural habitat in Analamazaotra **G***Ravenala madagascariensis*, the Traveler’s Palm (**B–G** photo J. Skejo).

Figs 3 , 4 Common name: Southern Devils’ pygmy grasshopper

Holocerus lucifer : Rehn (1929: 493; description, drawings, and distribution); Günther (1939: 91; reported from Antongil Bay and from Alahakato (= Lakato, approximately S19.171498, E48.510321)); Günther (1959: 10; included in key, reported sympatrically two Holocerus species; records from Antongil Bay and from Alahakato Forest); Günther (1974: 966–967; reported from Périnet and Rogez = Andasibe-Mantadia NP and Analamazaotra, but also from Antongil Bay, sympatrically with the other Holocerus species); Devriese (1991: 123–124; mentioned and depicted); Yin et al. (1996; listed in catalogue); Otte (1997; listed in catalogue); Cigliano et al. (2020; OSF catalogue);
Holocerus taurus : Skejo and Caballero (2016: Figs 2a, b; photographs of living specimens (an adult and a nymph), wrongly identified as H. taurus).

###### Derivatio nominis.

We name this neat new species in honor of Hendrik Devriese, a Belgian entomologist and botanist. Devriese is one of the world’s leading tetrigidologists, geographically specialized in African and Malagasy taxa, taxonomically in Tetriginae and Xerophyllini genera. He has hitherto introduced the terminology of pronotal carinae with an emphasis on Malagasy taxa, described five new genera, 12 new species, and one new subspecies, and many more are awaiting description, especially those from Madagascar. The specific epithet is a genitive case second Latin declension noun, derived from the Latin version of the surname ‘Devriese’ – N *devrieseus* G *devriesei*.

###### Type material.

(11 specimens: holotype and 10 paratypes): 1♂ HT Analamazaotra (S18.943 E48.428) 12.I.2010. leg. Miko (MNCN, Catalogue number MNCN_Ent 26936); (**1/10**) 1♀ PT, Madagascar, Tananarive, Lamberton 1914. (Catalogue number MNHN-EO-CAELIF 9070); (**2/10–3/10**) 2♀♀ PTs, Madagascar, Perinet, forêt côte Est, A. Seyrig, 1937. (Catalogue numbers MNHN-EO-CAELIF 9071, MNHN-EO-CAELIF 9072); (**4/10**) 1♀ PT, Madagascar, Forestier, Frappe, 1946. (Catalogue number MNHN-EO-CAELIF 9073); (**5/10–6/10**) 2♂♂ PTs, Madagascar, Perinet, forêt côte Est, A. Seyrig, 23-2-34. (Catalogue numbers MNHN-EO-CAELIF 9074, MNHN-EO-CAELIF 9075) (MNHN); (**7/10–9/10**) 2♂♂ + 1♀ Madagascar: Rogez, Madagascar Centr., I‐1932. A. Seyrig (Catalogue numbers MNCN_Ent 195226, MNCN_Ent 195227, MNCN_Ent 195230), (**10/10**) 1♀ PT (Catalogue number MNCN_Ent 268524) Madagascar: Rogez, Madagascar Centr., XII‐1931. A. Seyrig (MNCN).

###### Type material depository.

The holotype male is a wet preserved specimen, kept in 80% ethyl alcohol, deposited in MNCN Madrid. One hind leg of the holotype was isolated for future molecular studies. The paratypes are ten dry-mounted specimens. Four paratypes are deposited in the Orthoptera collection within the Entomological collections of the Museo de Ciencias Naturales, Madrid, while six paratypes are deposited in the Entomological collections of the Muséum national d’Histoire naturelle, Paris.

###### Additional material examined (altogether 17 specimens).

**Museum collections (9 specimens).** 2♂♂ Madagascar; Anovano, Madagascar (probably within hither Andasibe – Mantadia), I-1934. A. Seyrig (MNCN_Ent 195223, MNCN_Ent 195224) (MNCN); 2♀♀ Madagascar: Rogez (Analamazaotra), Madagascar Centr., II-1932. A. Seyrig (MNCN_Ent 195229, MNCN_Ent 195231) (MNCN); 1♀ Madagascar: Omalamazaotra (= Analamazaotra) I-1933 A. Seyrig (MNCN_Ent 195233); 1 nymph Madagascar: Omalamazaotra XII-1933. A. Seyrig (MNCN_Ent 195239); 4♀♀ Madagascar: Fito IV-V.1932. A. Seyrig (MNCN_Ent 195235, MNCN_Ent 195236, MNCN_Ent 195237, MNCN_Ent 195238).

**Online social media platforms (8 specimens).** 1♀ Moramanga region 25.VI.2011. obs. entomokot (Konstantin) (uploaded to iNaturalist); 1♂ nymph Andasibe-Mantadia NP: Andasibe 13.XI.2011. obs. P. Bertner (uploaded to Flickr); 1♂ nymph Vohimana reserve 29.XI.2013. obs. F. Vassen (uploaded to Flickr and Wikimedia Commons); 1♂ Ranomafana NP 12.V.2015. obs. Paul Bertner (uploaded to Flickr); 1♂ Andasibe-Mantadia NP: Périnet (Analamazaotra) 1.II.2018. obs. J.-Y. Grospas/ Biosphoto (uploaded to Alamy); 1♂ Analamazaotra (S18.943552 E48.428283) 18.I.2019. obs. Miko and Skejo (uploaded to iNaturalist); 1♂ Andasibe-Mantadia: Périnet (Analamazaotra) NP 26.III.2019. obs. J.-Y. Grospas/ Biosphoto (uploaded to Alamy); 1♂ Ranomafana NP obs. Marc Hoffmann (uploaded to Instagram).

###### Annotated specific diagnosis.

The new species, *Holocerus devriesei* sp. nov., is similar to *H. lucifer*, its only congener, but is easily distinguished from it by the following set of traits: (i) compared to *H. lucifer*, the new species *H. devriesei* sp. nov. has more robust (less elongated) femora of fore and mid legs; (ii) dorsal spines (elevated lateral carinae) are short and projected as acute triangular plates in *H. devriesei* sp. nov., whereas in *H. lucifer* they are long and decurved; (iii) middle prozonal spine (promedial projection) is sharp and higher in *H. devriesei* sp. nov. than in *H. lucifer*; and (iv) *H. devriesei* sp. nov. is generally a darker species, with less yellowish-green parts (*H. lucifer* is usually paler in coloration, but exceptions do occur). For a ***detailed description*** of *H. devriesei* sp. nov., see Rehn’s (1929) description of what he called ‘*H. lucifer*’.

###### Measurements.

Body length, pronotum length, pronotum width between the lateral lobes, maximum pronotum width (between the tips of the dorsal spines), and hind femur length are shown (Table 3).

**Table 3.** *Holocerus devriesei* sp. nov. measurements. Note that there are two measurements for pronotum width, one between the lateral lobes and the other between the dorsal spines.

	Body length	Pronotum length	Pronotum width (lateral lobes)	Pronotum width (dorsal spines)	Hind femur length
♂♂ (N = 6)	13.5–17.1 mm	19.8–22.9 mm	4.0–5.2 mm	6.2–7.2 mm	9.0–9.9 mm
♀♀ (N = 6)	18.0–23.2 mm	20.8–25.4 mm	4.9–6.1 mm	7.0–8.8 mm	10.2–11.4 mm

###### Distribution, habitat and threats.

*Holocerus devriesei* sp. nov. inhabits eastern Malagasy rainforests, from Ranomafana in the south, *via* Analamazaotra, rainforests around Lakato, Vohimana and Andasibe-Mantadia, all the way to the Antongil Bay, where it has an overlap in distribution with *H. lucifer*. The natural habitat of the species are primary and secondary rainforests, but on account of forest depletion and habitat degradation, population decline is expected, as well as extinction of its subpopulations in certain territories where the rainforest is undergoing degradation.

## Discussion and conclusions

**Figure 5.**
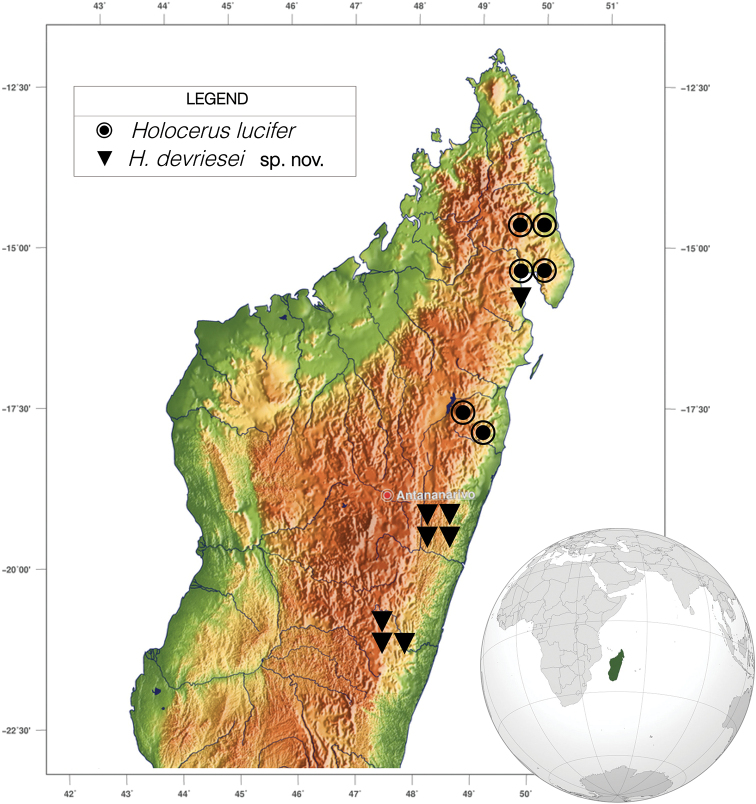
Distribution map of *Holocerus* species – *H. lucifer* (circles) and *H. devriesei* sp. nov. (triangles).

We know about the existence of two species of Malagasy pygmy devils within the genus *Holocerus*. A pale colored species with longer decurved spines (Figs 1, 2) inhabits the rainforests of Marojejy and Masoala, from around the Antongil Bay in the north to the Zahamena NP in the south (Figure 5), and should be referred to as *Holocerus lucifer*. A darker species with shorter angular spines (Figs 3, 4), which should from now on be referred to as *H. devriesei* sp. nov., inhabits the rainforests from Ranomafana in the south to the Bay of Antongil in the north (Figure 5). There are distribution overlaps between the two species, but as there are no evidences of hybridization, we treated them as separate species.

The name confusion originated when Rehn (1929) described the pale-colored species with longer spines (the true *H. lucifer*) as a new species under the name *H. taurus*, while at the same time applying the name ‘*lucifer*’ to designate the darker specimens with shorter and angular spines (*H. devriesei* sp. nov.). Subsequent authors followed Rehn’s application of these names (Günther 1939, 1959, 1970; Yin et al.1996; Otte 1997). This was a serendipitous error, as Rehn (1929) did not have a possibility to check what Serville (1838) described under the name *H. lucifer*. As we had the name-bearing specimens of both species before us, it became clear that *H. taurus* syn. nov. represents a synonym of *H. lucifer*. This confusion was furthermore inflamed by Skejo (Skejo and Caballero 2016; Skejo 2017) who applied the epithet ‘*taurus*’ to a dark species with angled spines. Now, with the description of this dark *Holocerus* species as *H. devriesei* sp. nov., we hope that this nomenclatural knot has been untangled and that both experts and citizen scientists will be able to correctly name *Holocerus* specimens. The IUCN Red List assessments (Danielczak et al. 2017a, b) should be amended accordingly.

## Supplementary Material

XML Treatment for Holocerus

XML Treatment for Holocerus lucifer

XML Treatment for Holocerus devriesei
